# Correlated Evolution between Mode of Larval Development and Habitat in Muricid Gastropods

**DOI:** 10.1371/journal.pone.0094104

**Published:** 2014-04-08

**Authors:** Paula Pappalardo, Enrique Rodríguez-Serrano, Miriam Fernández

**Affiliations:** 1 Odum School of Ecology, University of Georgia, Athens, Georgia, United States of America; 2 Departamento de Zoología, Facultad de Ciencias Naturales y Oceanográficas, Universidad de Concepción, Concepción, Chile; 3 Centro de Conservación Marina, Departamento de Ecología, Pontificia Universidad Católica de Chile, Santiago, Chile; Laboratoire de Biologie du Développement de Villefranche-sur-Mer, France

## Abstract

Larval modes of development affect evolutionary processes and influence the distribution of marine invertebrates in the ocean. The decrease in pelagic development toward higher latitudes is one of the patterns of distribution most frequently discussed in marine organisms (Thorson's rule), which has been related to increased larval mortality associated with long pelagic durations in colder waters. However, the type of substrate occupied by adults has been suggested to influence the generality of the latitudinal patterns in larval development. To help understand how the environment affects the evolution of larval types we evaluated the association between larval development and habitat using gastropods of the Muricidae family as a model group. To achieve this goal, we collected information on latitudinal distribution, sea water temperature, larval development and type of substrate occupied by adults. We constructed a molecular phylogeny for 45 species of muricids to estimate the ancestral character states and to assess the relationship between traits using comparative methods in a Bayesian framework. Our results showed high probability for a common ancestor of the muricids with nonpelagic (and nonfeeding) development, that lived in hard bottoms and cold temperatures. From this ancestor, a pelagic feeding larva evolved three times, and some species shifted to warmer temperatures or sand bottoms. The evolution of larval development was not independent of habitat; the most probable evolutionary route reconstructed in the analysis of correlated evolution showed that type of larval development may change in soft bottoms but in hard bottoms this change is highly unlikely. Lower sea water temperatures were associated with nonpelagic modes of development, supporting Thorson's rule. We show how environmental pressures can favor a particular mode of larval development or transitions between larval modes and discuss the reacquisition of feeding larva in muricids gastropods.

## Introduction

Modes of larval development of marine invertebrates are not homogeneously distributed in the ocean. The patterns of distribution of larval development have been described in relation to diverse factors ranging from latitude (or factors that vary with latitude, such as temperature or ocean productivity) to depth, substrate, trophic stability and adult size [1]–[7]. Several hypotheses concerning the selective pressures that could drive patterns of distributions of larval modes in the ocean have also been proposed and models analyzing the optimal strategies of larval development in a range of environmental parameters and oceanographic conditions have been developed [8]–[10]. This rich field of research, however, has simplified the complex scenario of environmental variables and the role of evolutionary relationships between species (but see [4]).

It is interesting that nonfeeding modes of development prevail at high latitudes, while pelagic feeding larvae predominate at lower latitudes [5],[7],[11],[12]. The prevalence of pelagic feeding larvae at low latitudes has been related to the effect of temperature on pelagic duration, which reduces survival rates at high latitudes [4],[12],[13]. But temperature alone does not explain the distribution of larval development in the ocean, since nonfeeding pelagic larvae, which are also subjected to increased mortality at low temperatures, are not affected in the same fashion [14]. On the other hand, the hypothesis that feeding larvae should be greatly affected at high latitudes due to the short duration of planktonic primary production [7] is well supported by the consistent increase in richness of species exhibiting nonfeeding larval development toward higher latitudes [4],[14]. However, the relationship between food availability, measured by proxies such as chlorophyll *a*, and the distribution of species with feeding larvae is less clear [4],[14]. Recently, it has been shown that the combination of low temperature and low ocean productivity explains the increase in the proportion of direct developers (nonfeeding, nonpelagic development) in marine invertebrates, suggesting a complex relationship between environmental factors and larval development [4]. In fact, other less studied factors, such as substrate type, also seem to affect the distribution of direct developing species [5],[15],[16] and therefore may (a) determine large scale patterns of distribution of species, and (b) explain exceptions to general patterns.

The impact of other environmental factors, not necessarily correlated with latitude, can help explain exceptions to expected patterns of species distribution [14]. The proportion of direct developers does not seem to change along latitude for shallow water gastropods in soft bottoms [5],[15], suggesting that habitat could be an important determinant of the distribution of larval development in the ocean. The association between direct development and soft bottom has also been suggested for polychaetes [17] and macroinvertebrates of sandy intertidal beaches [16]. The mechanisms to explain this relationship have been linked to patchiness and disturbance rates characteristics of soft bottom habitats [17],[18]. Brooding or reduced larval dispersal is expected to be favored in patchy habitats [18] and in disturbed soft bottoms, as it allows quick recolonization [17]. Indeed, groups exhibiting lecithotrophic development (pelagic nonfeeding larva) that inhabit soft bottom habitats also fail to fit the general latitudinal diversity pattern reported for most groups of marine and terrestrial organisms [14],[19]. Lecithotrophic development also tends to exhibit lower potential for dispersal [16]. The interplay between environmental factors, larval development, and potential for dispersal can shed light on our understanding of large scale patterns of distribution of life history strategies in the ocean.

A phylogenetic framework is necessary to help understand how the environment affects the evolution of larval types. Although most reported transitions in larval development of marine invertebrates are from feeding to nonfeeding development [20],[21], multiple origins of derived modes of larval development have been described in many groups [22]. The main purpose of this study is to evaluate the association between mode of larval development and habitat in muricid gastropods, considering two aspects of habitat: latitudinal distribution (and associated sea water temperature) of the species and type of substrate occupied by adults. Gastropods of the family Muricidae are distributed worldwide and exhibit a great diversity of larval development strategies [23]. We performed our analysis in a Bayesian phylogenetic framework, estimating the parameters of the model of traits evolution across independent samples of trees [24]. Additionally, Bayesian methods applied to the ancestral reconstruction of traits allow us to combine information on the uncertainty of the presence of a node with the information of the estimate of the ancestral character state for that node [25]. We provide evidence of correlated evolution between larval mode of development and the two aspects of habitat analyzed, and suggest a possible evolutionary route through which a change in type of substrate occupied by adults can produce a transition in type of larval development.

## Methods

### Biological data set

We conducted an intensive literature search to compile information on taxonomy, latitudinal distribution, sea water temperature, type of substrate occupied by adults and mode of larval development in marine gastropods of the Muricidae family. Main preferred substrate for each species was classified as soft (sand, mud, seagrass) or hard (rock, coral reefs, calcareous) bottom. We simplified the classification of larval development [18] according to feeding type and larval stage in three categories: (a) planktotrophic (pelagic, feeding larva), (b) lecithotrophic (pelagic, nonfeeding larva), and (c) direct developers (nonpelagic, nonfeeding development). All species within this family encapsulate embryos, which after a period of benthic development inside the capsules hatch either as larvae (that continue development in the plankton) or crawl away as juveniles [23]. Extraembryonic sources of nutrition can also be packed within the egg capsules in the form of intracapsular fluid or nurse eggs [26],[27]. We recorded the presence of nurse eggs, as it has been previously shown in calyptraeids gastropods that the transition from nonfeeding to a pelagic feeding larva could be more likely in clades with nurse eggs [28],[29]. Crossing both sources of information on larval development we developed our final classification of modes of larval development in the following four categories: 1) Planktotrophic, 2) Lecithotrophic larvae with nurse eggs, 3) Direct development with nurse eggs, 4) Direct development (including species without nurse eggs, or species that lack detailed information on presence/absence of nurse eggs). We classified 103 species of muricids into these categories, but we only found sequences in GenBank for 45; only information available until the end of September 2013 was included. Information on the species included in the phylogenetic analysis is detailed in Table S1 of the supplementary material; GenBank accession numbers are listed in Table S2.

If information on site of collection of egg capsules was available in the surveyed studies of larval development, we used the reported latitude of collection for the analysis (majority of the data, Table S1). When there was not clear relation between the study of intracapsular development and collection site, we performed the analysis using the midpoint of the latitudinal distribution range of the species described in the literature (31% of cases). Distributional ranges (degrees of latitude) were assumed to be continuous along the northern and southern ranges of the species. The majority of the species were from the Indowest Pacific, Eastern Pacific, and Western Atlantic but species from North and Eastern Atlantic, Western Pacific, Australia and New Zealand were also represented in the database (Table S1).

### Phylogenetic hypothesis

Nucleotide sequences of mitochondrial cytochrome oxidase I (COI, 657 bp), 16S rRNA (496 bp), 12S rRNA (1471 bp), and 28S rRNA (1451 bp) were obtained from GenBank for the 49 species analyzed in this study (45 muricids and 4 outgroups, Table S2). When there was more than one sequence listed, we chose the most informative sequence. Four outgroups were selected belonging to related neogastropod families: Buccinidae, Conidae, Melongenidae and Nassaridae [30]. The four genes selected were not equally available for all taxa. Therefore, our final database included: 92% COI sequences, 73% of 16S rRNA, 69% of 12S and 71% of 28S rRNA for the 49 species analyzed. Even though a 24% of our species were represented only by one gene, we included them since our results agreed with the current taxonomy for the family and published phylogenies [31]. Each gene was aligned separately with MUSCLE [32] using the default settings, and further optimized by eye using MEGA [33]. After the alignment, all genes were combined in Mesquite [34].

The combined dataset was analyzed with a general likelihood-based mixture model of gene sequence evolution which considers rate and pattern heterogeneity in the data [35]. Using this approach, no prior knowledge for partition of the data is needed, and a variety of possible models of evolution and parameters can be performed. This model was implemented using Markov Chain Monte Carlo methods within a Bayesian framework (BMCMC), using BayesPhylogenies 1.1 (http://www. evolution.rdg.ac.uk/BayesPhy.html) [35]. We ran three independent BMCMC analyses, using 37,370,000 generations of phylogenetic trees, sampling every 10,000 trees to assure that successive samples were independent. From the initial sample of 3,737 phylogenetic trees we removed the first 188 to avoid the inclusion of trees sampled before the convergence of the Markov Chain. To assess the stationary distribution of the Markov chain we visually inspected the log-likelihood values of the iterations of the Markov chain until it had reached convergence using the software Tracer v1.5 [36], checking for an effective sampling size higher than 500. Subsequently, we used the *acf* function of the R [37] package stats to conduct an autocorrelogram, subsampling with a lag of at least two cycles without significant autocorrelation. From this, we obtained a final sample of 102 independent phylogenetic trees, which were used for the comparative analyses.

### Data analysis

The evolution of continuous traits (latitude and associated sea water temperature) was analyzed with a generalized least squares model (GLS) [38],[39] implemented in the software BayesTraits, Continuous module [40]. To avoid the negative values of the southern hemisphere latitudes we re-scaled latitude considering the southernmost location as 1. We first analyzed which model describes the evolution of continuous variables better by comparing the standard GLS model (which assume that traits evolve under random walk) and the directional GLS models, to then select the best model using Bayes factor. This allowed us to evaluate directional trends in the evolution of traits. Then, we evaluated phylogenetic signal for each trait using the sample of trees obtained from the Bayesian analysis. For continuous traits we estimated the three phylogenetic parameters defined by Pagel [39],[41]: 1) lambda, 2) kappa, and 3) delta.

The scaling parameter lambda (λ) evaluates if the evolution of traits is predicted by phylogeny by assessing the similarity in the traits between species. It then verifies the principal assumption for the use of the Comparative Method, if λ = 0 a phylogenetic correction is not necessary because evolution of traits is independent of the phylogeny [41],[42]. If λ<1, traits are less similar among species than expected according to the phylogeny; if λ = 1 the traits are evolving as expected by the sample of trees used. The branch length scaling parameter kappa (κ) distinguishes between punctuated or gradual evolution. It takes values of 1 when trait evolution is proportional to branch length, supporting a gradual mode of evolution and of 0 when trait evolution is independent of branch length, suggesting a punctuational mode of evolution [41]. The path-length scaling parameter delta (δ), contrasts adaptive radiation versus species adaptation. A value of δ = 1 suggests constant rate of evolution, δ<1 indicates adaptive radiations (because traits change rapidly first and then remains stable) and, δ>1 suggests that longer paths contribute more to trait evolution, indicating that recent evolution is more important [41]. These parameters were forced to 0 and 1, and then compared with observed values using Bayes factors.

To analyze the evolution of larval development we used the most recent common ancestor approach [25] in a BMCMC framework to infer the ancestral character states in the root, and in several nodes of interest. We performed the analysis for the four categories of larval development described above, and also estimated the ancestral character states in the root for type of substrate and larval development coded as a binary variable in two different ways: (1) pelagic (planktotrophic and lecithotrophic) or nonpelagic (direct developers), and (2) feeding (planktotrophic) or nonfeeding (lecithotrophic and direct developers). This analysis was performed in the software BayesTraits, Multistate module [40]. The presence of phylogenetic signal in larval development was evaluated measuring the probability of shared trait values between closely related taxa, using the Bayesian Tip-association Significance Testing Software (BaTS) [43]. This software allows estimation of phylogeny-trait correlations in discrete traits from the posterior distribution of trees obtained in the Bayesian analysis. We estimated the parsimony score, the association index and the monophyletic clade size statistic [43]. Lower parsimony score and association index values represent strong phylogeny-trait association, while the monophyletic clade size statistic increases with the strength of the phylogeny-trait association.

In order to evaluate correlated evolution between mode of larval development and type of substrate occupied by adults we used larval development coded as a binary variable (described above), which allowed us to apply Bayesian methods developed for discrete binary traits [44],[45]. First, we evaluated if evolution between traits matched an independent or dependent model of evolution. The test basically compares the marginal likelihood of a model where transition rates of each variable are independent of the state of the other (independent model) versus a model where rates of change depend on the state of the other variable (dependent model). The transition rates between states are indicated by the parameter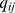, that represents the rate of transition from state *i* to state *j*, where *i* is larval development and *j* the type of substrate occupied by adults. When dependent models were chosen, we ran a reversible-jump Markov chain Monte Carlo in BayesTraits, Discrete module [40] to search among the whole range of possible dependent models. As reversible-jump Markov chain simultaneously estimates the posterior distributions of the rate parameters in the model of trait evolution, it allowed testing alternative evolutionary scenarios to explain the observed data. Using a sample of phylogenetic trees, this method also incorporates phylogenetic uncertainty.

Finally, and in order to analyze the relationships between mode of larval development and sea water temperature, we performed a phylogenetic logistic regression with Firth correction using the function PLogReg.m [46] created for Matlab. This method was developed to analyze the relationship between a binary dependent variable (0 or 1) with non-independent values among species and independent variables that can be binary or continuous [46]. We used larval development coded as presence of pelagic or feeding larva (0: yes, 1: no) as the dependent variable. Temperature was log transformed and standardized to have means equal to 0 and standard deviations equal to 1, so that the magnitude of regression coefficients represents effect sizes [46]. The variance-covariance matrix to run the phylogenetic regressions was obtained in the module PDAP of Mesquite, using the consensus of the trees sampled.

## Results

### Phylogenetic reconstruction

**Figure 1:**
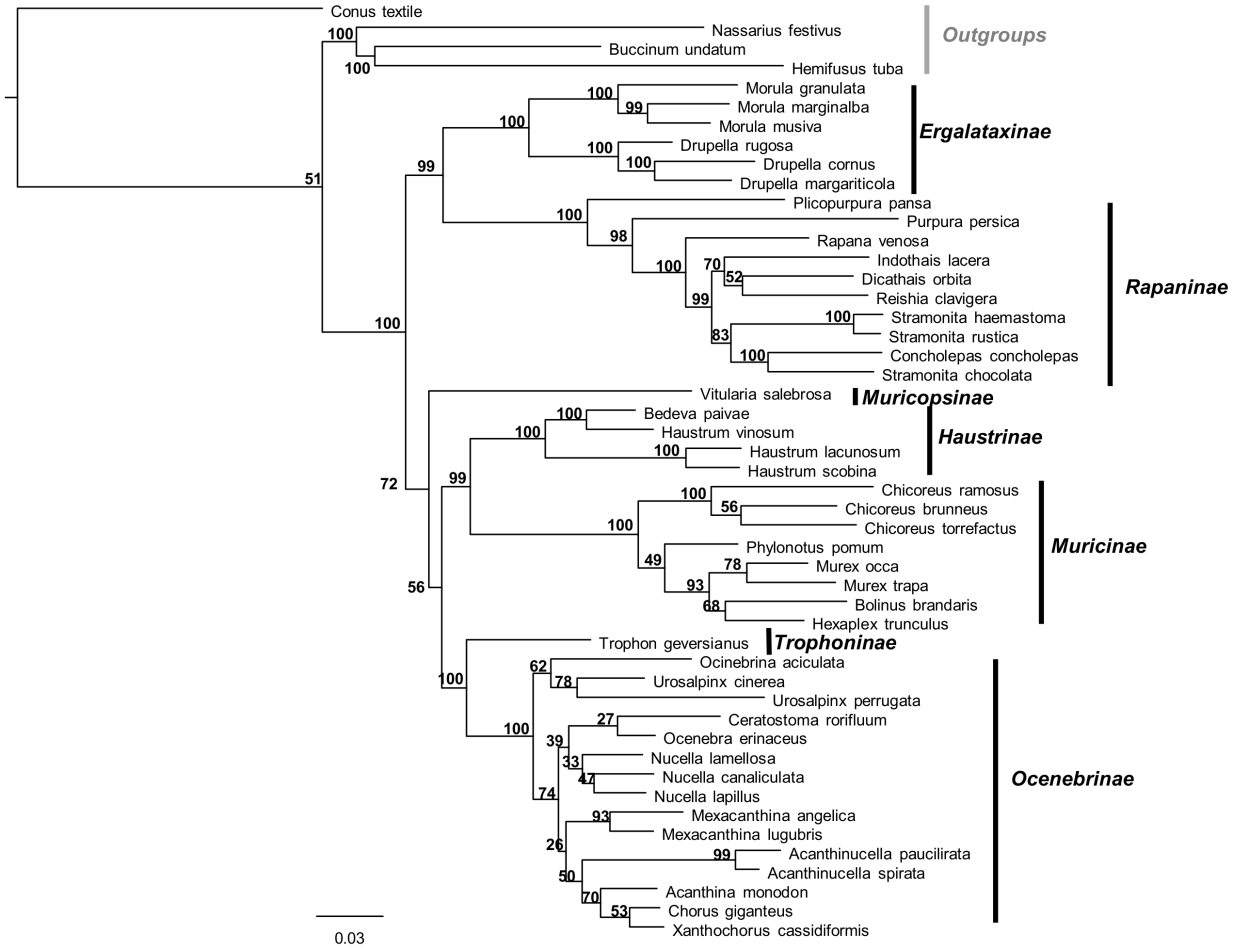
Phylogenetic reconstruction of muricid gastropods based on COI, 16S rRNA, 12S rRNA and 28S rRNA. The consensus tree was obtained through a Bayesian approach, using a general likelihood-based mixture model of gene sequence evolution implemented with Markov Chain Monte Carlo methods. Numbers above the nodes indicate the posterior probability of occurrence for each clade.

The 45 muricid species included in this study belong to 7 of the 10 subfamilies proposed for the Muricidae family. The consensus tree topology obtained from the sample of 102 trees was rooted using *Conus textile* (Fam. Conidae); the other outgroups selected were *Buccinum undatum* (Fam. Buccinidae), *Hemifusus tuba* (Fam. Melongenidae) and *Nassarius festivus* (Fam. Nassaridae). We observed in the consensus tree that both the Muricidae family and the proposed subfamilies (Muricinae, Ergalataxinae, Rapaninae, Haustrinae and Ocenebrinae) represent well-supported monophyletic groups (Fig. 1). The subfamilies Muricopsinae and Trophoninae were represented only by one species (*Favartia ponderi* and *Trophon geversianus*; Fig. 1). Our phylogenetic reconstruction agrees with a recent molecular phylogeny of muricids [31],[47] supporting its use for the comparative analysis.

### Trait evolution and phylogenetic signal

In the muricids studied, the evolution of sea water temperature was better explained by a directional model, while the random-walk model was the best predictor for latitude (Table 1). The directional change parameter for temperature showed that the dominant direction of evolutionary change was toward species inhabiting regions in warmer temperatures (Table 1). The random-walk model selected for latitude implies no particular trend in the evolution of this trait coded as a continuous variable. Both latitude and temperature were influenced by phylogeny and the estimated phylogenetic signal was different from zero (Table 2), which justified the use of comparative methods. A gradual evolution was observed in both traits (0<κ<1, Table 2); occurring at a constant rate δ = 1 in latitude and with values δ>1 for temperature, suggesting a higher contribution of longer paths to evolution of this trait.

**Table 1:** Parameters that describe the models of evolution analyzed for latitude and sea water temperature in muricid gastropods and criterion for selection.

Trait	Model of evolution	Alpha (α)	Beta (β)	Bayes Factor
Sea water temperature	Random walk	18.46 (11.15; 25.42)	-	
	Directional*	1.14 (−29.21; 20.61)	67.02 (23.18; 99.97)	−13.02
Latitude	Random walk*	75.00 (48.87;100.0)	-	
	Directional	67.32 (14.37; 99.72)	6.32 (−43.47; 99.82)	−0.50

The high probability distribution for parameters alpha and beta is shown within brackets. The selection of evolutionary models that best fit the evolution of the variables was made by Bayes Factors implemented in Tracer v.1.5 software [36]. Asterisks indicate the models selected.

**Table 2:** Pagel's [39],[44] scaling parameters for latitude and sea water temperature in muricid gastropods and criterion for selection.

Trait	Parameters	Value	Bayes Factor
**Temperature**	**Lambda λ**		
	λ Estimated*	0.63 (0.22; 0.93)	
	λ Forced = 1		0.03
	λ Forced = 0		0.09
	**Kappa κ**		
	κ Estimated*	0.95 (0.53; 1.31)	
	κ Forced = 1		−0.31
	κ Forced = 0		−0.42
	**Delta δ**		
	δ Estimated *	1.57(0.83; 2.50)	
	δ Forced = 1		−0.06
**Latitude**	**Lambda λ**		
	λ Estimated	0.90 (0.67; 0.99)	
	λ Forced = 1*		8.81
	λ Forced = 0		0.01
	**Kappa κ**		
	κ Estimated *	0.29 (0.00; 0.66)	
	κ Forced = 1		−1.61
	κ Forced = 0		−1.47
	**Delta δ**		
	δ Estimated	0.81 (0.13; 1.54)	
	δ Forced = 1 *		1.60

Parameters were selected using the Bayes Factor (BF) implemented in Tracer v.1.5 software [36]. Asterisks indicate the models selected.

**Figure 2:**
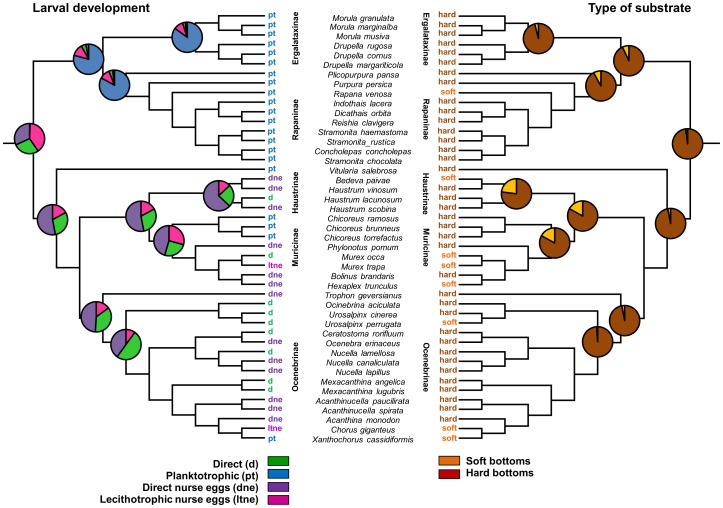
Reconstruction of the ancestral state of mode of larval development and the type of substrate occupied by muricid gastropods. Reconstruction was made using the most recent common ancestor approach [25], based on the topology and branch length obtained in the sample of trees. The posterior probability of occurrence of each state is represented in the main nodes. Larval development is classified as direct, direct with nurse eggs, lecithotrophic with nurse eggs and planktotrophic; the type of substrate is classified as soft or hard. The character states present in extant species are represented in the tips of the tree. The name of Muricids subfamilies are indicated in each clade except for the two subfamilies with only one species included in our study (*Vitularia salebrosa*, Muricopsinae; *Trophon geversianus*, Trophoninae).

The ancestral character state estimation for mode of larval development and type of substrate showed that the most probable common ancestor of the muricids had nonswimming and nonfeeding larva, and lived on hard bottoms (Fig. 2, Table 3). Hard bottom was the ancestral state estimated with high probability for the root and all the subfamilies (Fig. 2, Table 3). From this ancestor, planktotrophy evolved three times, in the Muricinae, Ocenebrinae, and the ancestor of Ergalataxinae, Rapaninae and Muricopsinae (Fig. 2). When larval development was considered as a four state variable to include the presence/absence of nurse eggs, the ancestral character state estimation for the root indicates high combined probability of nurse eggs presence (0.39 for lecithotrophic development and 0.32 for direct development; Fig. 2 Table 3).

**Table 3:** Posterior probabilities of the ancestral state reconstruction of traits in the nodes that give rise to the principal subfamilies of muricid gastropods.

Trait	State	Root	Muricinae	Ergalataxinae	Rapaninae	Haustrinae	Ocenebrinae
**Larval development**	Planktotrophic	0.01±0.002	<0.00±0.000	**0.85±0.003**	**0.83±0.004**	<0.00±0.000	<0.00±0.000
	LecithotrophicNE	**0.39±0.011**	0.29±0.007	0.10±0.0031	0.11±0.003	0.13±0.003	0.10±0.004
	Direct	0.28±0.006	0.25±0.006	0.03±0.002	0.04±0.002	0.24±0.005	**0.49±0.012**
	DirectNE	0.32±0.010	**0.45±0.010**	0.02±0.001	0.02±0.001	**0.63±0.006**	0.40±0.012
**Feeding larva**	Yes	0.07±0.006	0.02±0.002	**0.98±0.000**	**0.97±0.000**	<0.00±0.000	0.00±0.000
	No	**0.93±0.006**	**0.98±0.002**	0.02±0.000	0.03±0.000	**>0.99±0.000**	**1.00±0.000**
**Pelagic larva**	Yes	0.03± 0.004	0.13±0.002	**0.97±0.000**	**0.95±0.001**	<0.00±0.000	0.00±0.001
	No	**0.97± 0.004**	**0.99±0.002**	0.03±0.000	0.04±0.001	**>0.99±0.000**	**1.00±0.001**
**Substrate**	Hard	**0.98± 0.002**	**0.83±0.004**	**0.96±0.002**	**0.92±0.003**	**0.77±0.003**	**0.99±0.001**
	Soft	0.02± 0.002	0.17±0.004	0.04±0.002	0.08±0.003	0.23±0.004	0.01±0.001

Traits analyzed were mode of larval development (NE: nurse eggs), type of development coded as presence of pelagic or feeding larva, and type of substrate occupied by adults (soft or hard bottoms). Posterior probability of occurrence of traits is indicated ± standard error; with the higher values for a given trait indicated in bold.

All classifications of mode of larval development and type of substrate showed phylogenetic signal, measured with the parsimony score and the association index (Table 4). For all traits, the parsimony score and the association index were lower than expected under a null distribution of taxon–character (P-value of the significance test is shown in Table 4). The monophyletic clade size statistic is calculated for each state of character, and the significance test assesses if the values are higher than expected under the null distribution, which indicates stronger phylogenetic trait associations [43]. Our results showed strong phylogeny-trait associations for direct developers with nurse eggs and planktotrophic species, for both types of swimming larvae, for feeding larva and for soft bottom dwellers (Table 4).

**Table 4:** Phylogenetic signal for mode of larval development in muricid gastropods.

Trait	Statistic	BaTS estimate (95% HPD CIs)	P-value
**Larval development**	AI	1.49 (1.11, 1.19)	**0.00**
	PS	12.39 (11, 14)	**0.00**
	MC (Planktotrophic)	16.09 (16, 17)	**0.01**
	MC (LecithotrophicNE)	2.31 (2, 3)	0.69
	MC (Direct)	1 (1, 1)	1.00
	MC (DirectNE)	2.62 (2, 3)	**0.02**
**Pelagic larva**	AI	0.60 (0.36, 0.84)	**0.00**
	PS	5.88 (5, 7)	**0.00**
	MC (Pelagic)	16.10 (16, 17)	**0.01**
	MC (Nonpelagic)	4.87 (4, 7)	**0.01**
**Feeding larva**	AI	0.35 (0.15, 0.51)	**0.00**
	PS	4.33 (4, 5)	**0.00**
	MC (Feeding)	16.10 (16, 17)	**0.01**
	MC (Nonfeeding)	5.10 (4, 7)	0.08
**Substrate**	AI	1.24 (0.91, 1.57)	**0.02**
	PS	8.00 (7, 9)	**0.02**
	MC (Hard bottoms)	7.07 (7, 7)	0.25
	MC (Soft bottoms)	2.55 (2, 3)	**0.02**

Mode of larval development (NE: nurse eggs), type of development coded as presence of pelagic or feeding larva, and type of substrate occupied by adults estimated using Bayesian Tip-association Significance testing software (BaTS) [41]. AI: association index; PS: parsimony score; MC: monophyletic clade size statistic; HPD CIs highest posterior density confidence intervals. P-values from the BaTS null hypothesis test lower than 0.05 indicated in bold.

### Correlated evolution

The evolution of larval development was not independent of the evolution of habitat (type of substrate occupied by adults), there was significant correlated evolution. The dependent model of evolution was selected for both classifications of mode of larval development (coded as presence of pelagic or feeding larva). The selection of evolutionary models that best fit the evolution of the variables was made by Bayes factors implemented in Tracer v.1.5 software [36]. The Bayes factors for the dependent models were 63.60 for habitat versus pelagic stage, and 31.74 for habitat versus feeding larva.

**Figure 3:**
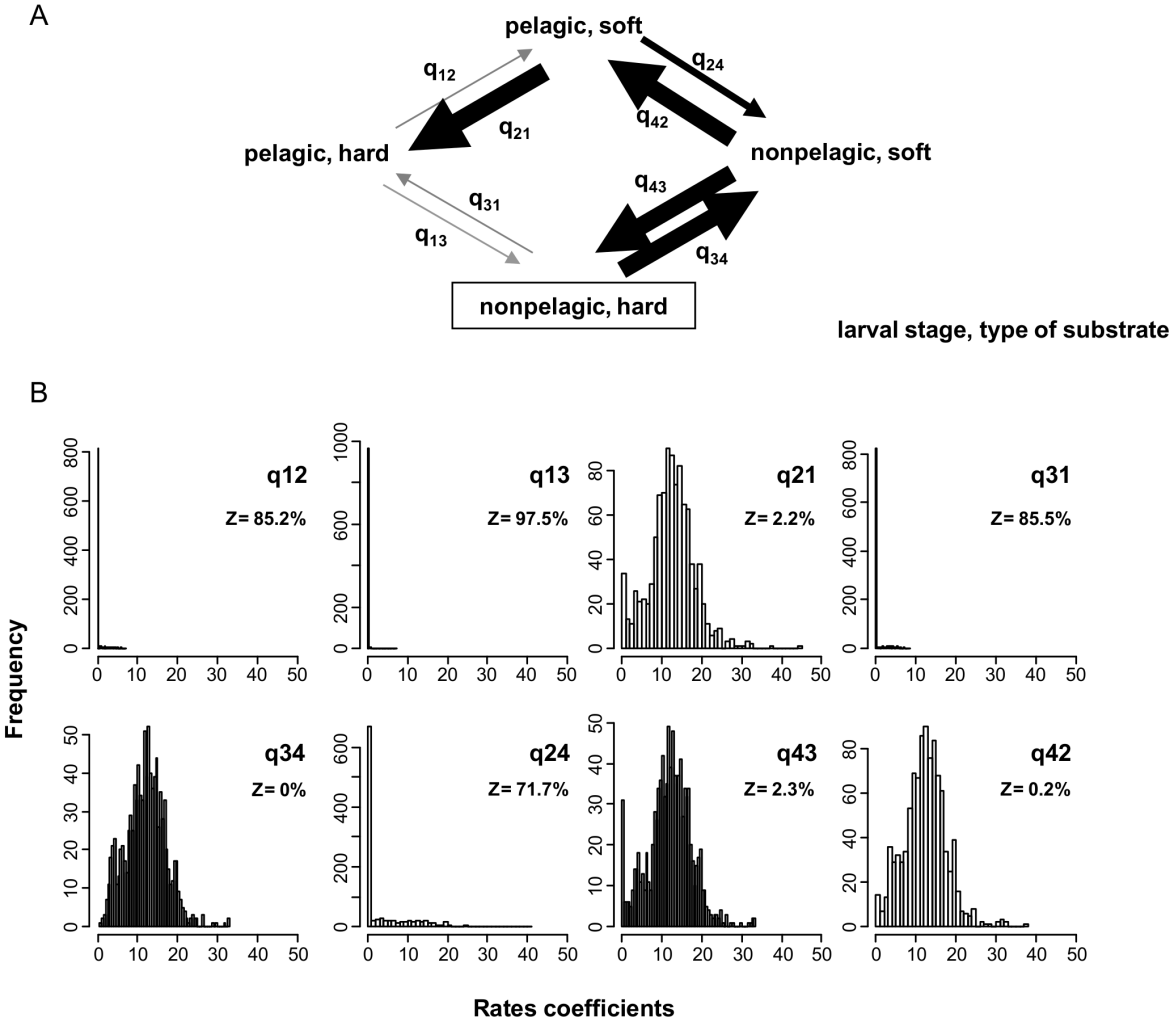
Evolutionary pathways and rates coefficients of the model of dependent evolution between larval development and type of substrate occupied by adults. A) Flow diagram describing evolution between each possible combination of larval stage and type of substrate (transition rates are represented proportional to the average values), B) Posterior distributions of the rate coefficients of the model. The plots are arranged so that vertical pairs correspond to rates that must be the same for the independent model to be true [45]. Z indicates the proportion of time the rates are assigned to zero.

The average transition rates of the model of dependent change were used to represent the evolutionary pathways that describe evolution between larval stage and habitat in our data (Fig. 3A); the model consisted of eight parameters 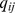 representing the rate of transition from state *i* to state *j*, where *i* and *j* represented combination of pelagic stage of larvae and type of substrate occupied by adults. The posterior distribution of the rate coefficients of this model explains why the dependent models were selected (Fig. 3B). Significant differences between the individual rates in pairs (*q_12_*, *q_34_*) and (*q_31_*, *q_42_*) explained the prevalence of models of correlated evolution in the posterior sample. We observed that *q_12_* was in the zero bin 85.2% of the time, while *q_34_* was never zero (Fig. 3B). Similarly, *q_31_* was in the zero bin 85.5% of the time, and *q_42_* only 0.2% of the time (Fig. 3B).

The evolutionary pathways (Fig. 3A) showed that from the estimated ancestral character state of nonpelagic development in hard bottoms, the type of substrate changed first (*q_34_*>*q_31_*), which could generate a selective pressure that favored a change in pelagic stage from nonpelagic to pelagic (*q_42_*>0). From a pelagic larva in soft bottoms a change in habitat back to hard bottoms was the most probable route (*q_21_*>0). Differences between rates *q_13_*/*q_31_* and *q_24_*/*q_42_* indicated that in hard bottoms the change between pelagic stages is never favored (*q_13_* = *q_31_*≈0), while in soft bottoms the change between pelagic stages can occur, and the transition rates are higher for the transition nonpelagic to pelagic (Fig. 3A). We only showed the results for pelagic stage of larva, because the posterior distributions of the rate coefficients were almost identical for feeding stage of larva.

**Figure 4:**
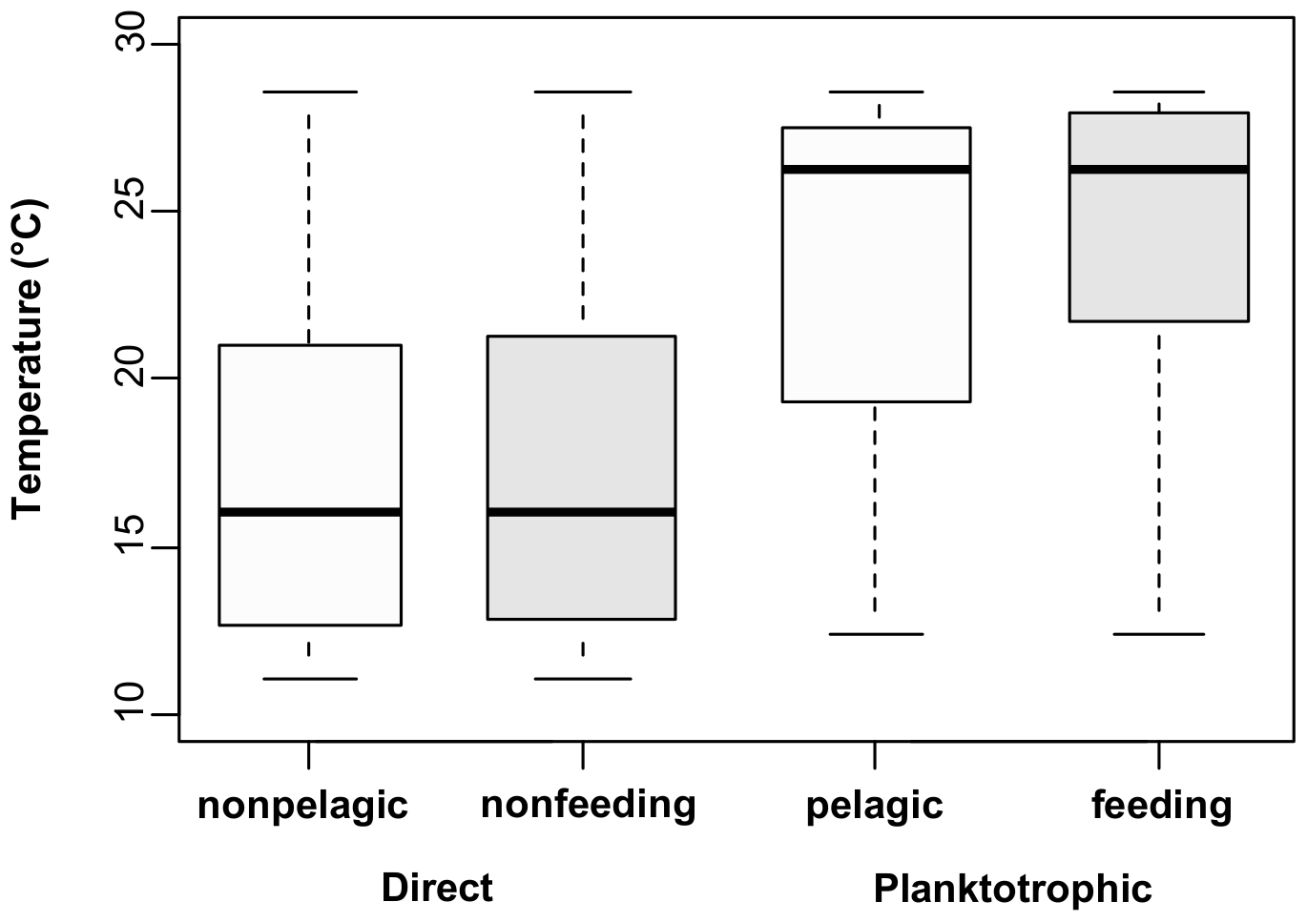
Relationship between temperature and type of larval development. The box plots show the sea water temperature associated with each mode of larval development for pelagic stage (light gray) and feeding type (darkgray). The horizontal band indicates the median, the bottom and top of the box delimit the first and third quartiles and the whiskers point the values within 1.5 times the interquartile.

The phylogenetic logistic regression performed to evaluate if the evolution of larval development is independent of the evolution of sea water temperature showed that there was correlated evolution between these two variables. Temperature had a significant effect on pelagic stage of larva; the negative coefficient of the regression showed that lower temperatures were associated with nonpelagic mode of development (Fig. 4, Table 5). Nonfeeding development showed a similar trend but the probability was only marginally significant. The values of phylogenetic signal (*a*) associated with the logistic regression were not significant (Table 5). However, phylogenetic logistic regression is still recommend instead of conventional logistic regression, because when phylogenetic signal is strong it has been shown that this analysis has little power to detect phylogenetic signal in the residual variation [46].

**Table 5:** Estimated parameters of the phylogenetic logistic regressions performed to assess the effects of sea water temperature on the presence of pelagic stage or feeding larva in muricid gastropods.

	Parameter	Estimate	Bootstrap mean	Boostrap confidence interval	P-value
**Feeding larva**					
Temperature	*a*	0.19	0.19	(−3.99, 3.99)	0.098
	*b_0_*	−0.17	−0.17	(−2.35, 2.23)	0.868
	*b_1_*	−0.64	−0.66	(−1.81, −0.07)	0.087
**Pelagic larva**					
Temperature	*a*	−0.11	−0.11	(−3.99, 3.99)	0.097
	*b_0_*	−0.33	−0.32	(−2.37, 1.80)	0.779
	*b_1_*	−0.76	−0.78	(−1.90, −0.01)	0.045

The response variable was coded as 0: present, 1: absent. Phylogenetic logistic regression with Firth correction using the function PLogReg.m [46] created for Matlab. Parameter estimates for phylogenetic signal (*a*) and for the regression coefficients (*b_0_, b_1_*). P-values lower than 0.05 indicated in bold. Bootstrapping was performed by simulating 2000 data sets.

## Discussion

Since mode of larval development can affect evolutionary processes and ultimately, species richness [48]–[50], it is important to understand the advantages and disadvantages of each mode [51], how environmental conditions might influence this trait and more importantly, the transitions between larval types. We demonstrated the association between mode of larval development and habitat in a group of marine gastropods belonging to the Muricidae family. Our results show that the most probable ancestor of the muricids had nonpelagic (and nonfeeding) development and lived in hard bottoms. We showed a possible evolutionary route whereby a change in type of substrate occupied by adults can produce a transition in larval development and how these transitions are only common in lineages inhabiting soft bottoms. Sea water temperature was also important to explain the evolution of larval development; as the decrease in temperature towards the pole has been proposed to explain the decrease in pelagic development toward higher latitudes (Thorson's rule) [7], the observed association between lower temperatures and nonpelagic modes of development provides support for this explanation. We will discuss if the reacquisition of feeding larva that we observed in our data could support the mechanism proposed to explain this transition in other gastropods.

Parallel changes in larval development are common and these changes can occur quickly [22], which affects the confidence in reconstruction of ancestral character states. Cunningham [52] discussed the problems of parsimony methods of ancestral character reconstruction to evaluate some evolutionary hypotheses and mentioned some advantages of maximum likelihood methods such as providing estimation of support. The Bayesian methods that we applied to the ancestral reconstruction not only provide estimation of support but also allow us to combine information on the uncertainty of the presence of a node (by using a sample of trees) with the reconstruction of the ancestral character state for a particular node [25]. A recent molecular phylogeny of Neogastropoda placed Muricidae as a sister group of families exhibiting predominantly lecithotrophic or direct larval development such as Buccinidae and Melongenidae [30],[53], which is in line with our findings of a nonfeeding/nonpelagic ancestral state for muricids. In contrast, in the basal groups of Neogastropoda, such as tonnoideans and cypraeoids, planktotrophic development is widespread. Despite the fact that transitions from feeding to nonfeeding are more common in marine invertebrates, in some groups feeding larvae have been reacquired [20],[21],[28]. The transitions between types of larval development have important evolutionary consequences, since this trait can affect the rates of diversification of clades [48],[54].

The potential barrier to reacquisition of feeding larva is the loss of the complex structures used for feeding, as exemplified by echinoderms [21]. However, observations of the intracapsular development of calyptraeid gastropods show that the structures used for feeding by planktotrophic species are also present in the intracapsular development of some direct developers with nurse eggs [28],[29]. The reacquisition of feeding larva could be achieved in direct developing species with nurse eggs because velar structures are still functional for ingestion of small particles or manipulation of nurse eggs [55], although other uses of the velum such as assimilation of intracapsular albumen are also described in littorinas [56]. Our findings of planktotrophic development re-appearing in muricids within clades of species with nurse eggs (e.g. *Xanthochorus cassidiformis*) or from ancestors with a high probability of nurse egg presence (e.g. the subfamily Muricinae) suggest that the same explanation could be applied to muricids. In fact, intracapsular observations of embryos of *Acanthina monodon* (direct developer with nurse eggs, P.P. personal observations) and *Chorus giganteus* (lecithotrophic development with nurse eggs) [57] show velar structures similar to *Xanthochours cassidiformis* (planktotrophic development) [58]. Detailed observations that explore the function of the velar structures during intracapsular development in these muricids could help to clarify this issue.

**Figure 5:**
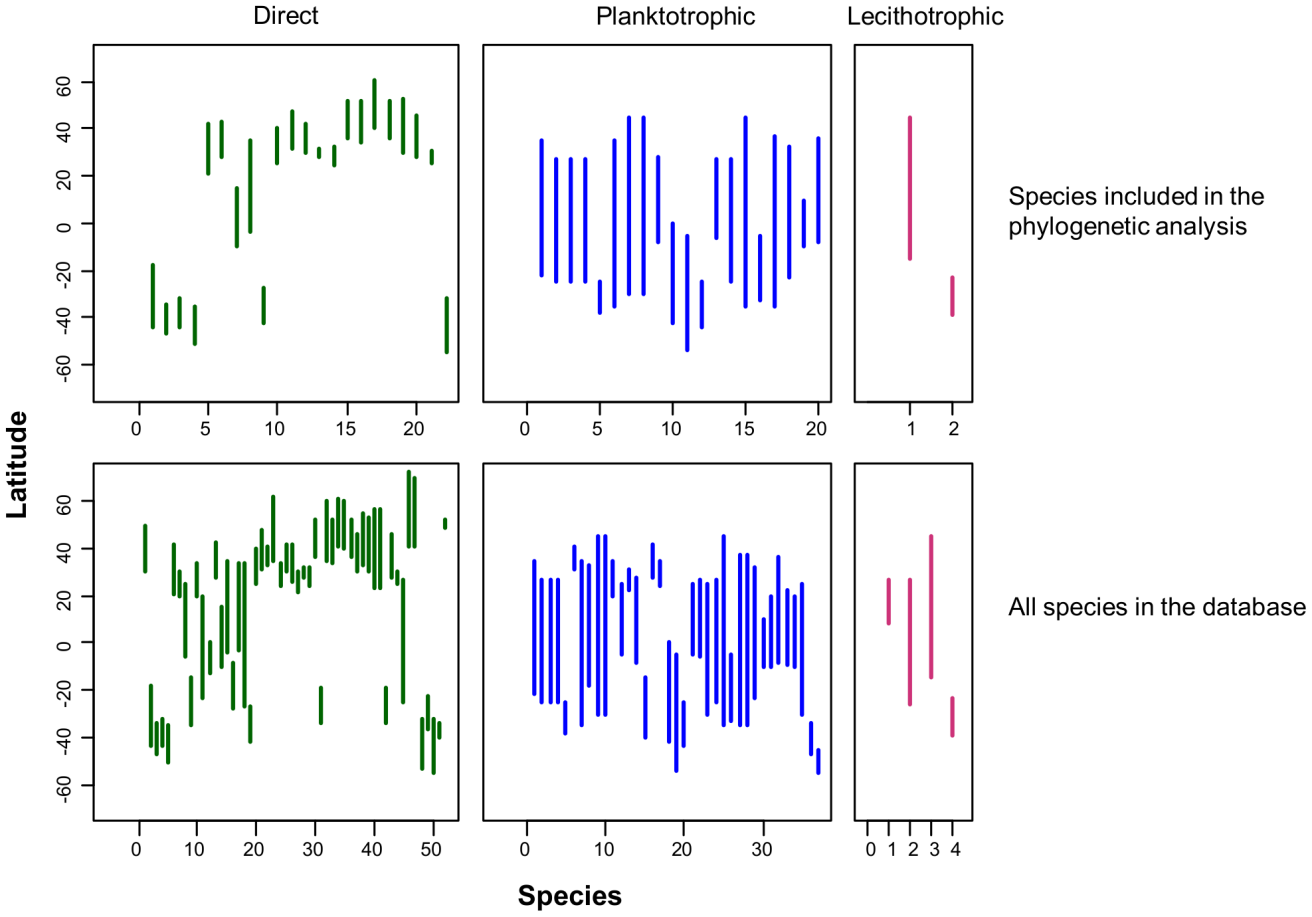
Latitudinal distribution of modes of larval development. The latitudinal distribution for species with planktotrophic larvae (blue), lecithotrophic larvae (purple) and direct development (green) is shown using vertical lines from the northern to the southern point of distribution reported in the literature. The panels show the species included in the phylogenetic (upper) and all the species present in our database (lower).

The correlated evolution found between temperature and larval development supports Thorson's rule, and highlights that in groups where larval development is not fixed, the environment can exert selective pressures on larval strategies. By incorporating phylogeny, we showed that the environment is a major determinant of larval development in muricids gastropods (i.e., developmental mode is not simply a function of phylogenetic constraint). Extant species of Muricidae inhabit warmer temperatures than their ancestors, and we showed that warmer temperatures are associated with a higher proportion of species with planktotrophic development. This suggests that planktotrophic development is advantageous in this environment [7],[13], assuming that food is not in shortage [3]. The latitudinal patterns of distribution of the muricids included in the phylogeny and the other muricids in our database confirm this (Fig. 5). This analysis provides a different perspective from the traditional approach explaining the loss of planktotrophy at high latitudes and it also highlights the advantages of planktotrophic development which is regained as the group diversified from high to low latitudes.

There are general benefits of planktotrophic development such as higher fecundity and dispersal away from parents that could help to avoid competition for resources and to decrease inbreeding in the next generation [51]. In addition, the decrease in pelagic larval duration at warmer temperatures offers at least two more advantages to pelagic larvae. One is decreased mortality, by reducing the time that pelagic larvae are exposed to different mortality sources [13]. The other is to reduce the time of exposure to currents and advection to unfavorable habitats [59]. On the other hand, nonfeeding modes of development are expected to be favored at high latitudes where higher mortality during extended larval development affects feeding larvae. These results agree with a recent study of latitudinal gradients of species richness of mollusks and crustaceans partitioned in modes of larval development, showing temperature as the main factor explaining species richness, but with opposite effects between feeding and nonfeeding larval types [14].

The type of substrate inhabited by gastropod species has been suggested to affect latitudinal patterns of larval development [5]. The correlated evolution between substrate type occupied by adults and mode of larval development is probably related to the different rates of dispersion that characterize each larval type. Low dispersal strategies seem to predominate in isolated soft sediment habitats such as sandy intertidal beaches or mudflats, which could be related to the advantages of staying in favorable, patchy habitats [16],[17]. Changes in type of substrate acted as a selective force that favored a change in larval development, although we did not find a unidirectional trend from pelagic to nonpelagic in soft bottoms as predicted; transitions from nonpelagic to pelagic stage were more frequent. Other factors related to the benefit of a pelagic dispersive phase, such as differences between mortality rates between the plankton and the benthos, or rates of habitat disturbance, could be responsible for the transitions in both directions [17],[51]. Isolated habitats in general, regardless of the type of substrate, could favor non pelagic modes of development, as suggested in oceanic islands for *Conus* gastropods [60].

Other environmental variables that could affect type of larval development are seasonality and ocean productivity. The fossil record of echinoids indicated that increasing seasonality could force a coordinated change in different clades toward non planktotrophic development [20]. Likewise, studies comparing larval development across the Panama isthmus have shown that nonfeeding development predominates in the Caribbean region, suggesting that lower productivity in this region is driving this pattern [3]. Accordingly, changes in productivity could also affect the evolution of the muricids. Diversification of the Rapaninae subfamily in the early Miocene apparently occurred during a time of high planktonic productivity in the tropics region [61], which might have favored transitions to planktotrophic development. Analysis of a clade with more species represented and a phylogeny accurately dated using fossils, could be used to evaluate hypotheses related to environmental changes in the past. Finally, we showed that transitions between modes of larval development are common in muricid gastropods, particularly in species inhabiting soft bottoms, but the mechanisms that caused transitions between larval types should be explored more by incorporating the role of isolation or patchiness.

## Supporting Information

Table S1 **Detailed information for the species used in this study.**
(PDF)

Table S2 **GenBank accession numbers for the species used in the phylogenetic analysis.**
(PDF)
