# Supplementary material for: Correlated Evolution between Mode of Larval Development and Habitat in Muricid Gastropods
Source: PLoS One. 2014 Apr 8;9(4):e94104. doi: 10.1371/journal.pone.0094104 (PMC3979742; doi:10.1371/journal.pone.0094104)
Supplement: Table S1 — Detailed information for the species used in this study. (PDF) [file pone.0094104.s001.pdf]

Table S1: Detailed information for the species used in this study.

Subfamily	Species name	Mode of larval development	Pelagic	Feeding	Nurse eggs	Habitat	Ocean	Latitude (-S)	SST	Point used
Outgroup	*Buccinum undatum*	DirectNE	no	no	present	soft	North Atlantic	58.5	9.55	site of collection
Outgroup	*Hemifusus tuba*	DirectNE	no	no	present	soft	Indowest Pacific	22.1	23.11	site of collection
Outgroup	*Nassarius festivus*	Planktotrophic	yes	yes	absent	soft	Indowest Pacific	32	19.355	site of collection
Outgroup	*Conus textile*	Planktotrophic	yes	yes	absent	hard	Indowest Pacific	5.47	27.92	site of collection
Ergalataxinae	*Drupella cornus*	Planktotrophic	yes	yes	absent	hard	Indowest Pacific	22.0	23.00	site of collection
Ergalataxinae	*Drupella margariticola*	Planktotrophic	yes	yes	absent	hard	Indowest Pacific	7.5	28.00	site of collection
Ergalataxinae	*Drupella rugosa*	Planktotrophic	yes	yes	absent	hard	Indowest Pacific	7.5	28.00	site of collection
Ergalataxinae	*Morula granulata*	Planktotrophic	yes	yes	absent	hard	Indowest Pacific	7.5	28.00	site of collection
Ergalataxinae	*Morula marginalba*	Planktotrophic	yes	yes	absent	hard	Australia	-33.4	21.70	site of collection
Ergalataxinae	*Morula musiva*	Planktotrophic	yes	yes	absent	hard	Indowest Pacific	22.0	23.00	site of collection
Haustrinae	*Bedeva paivae*	DirectNE	no	no	present	soft	Indowest Pacific	-32	21.52	site of collection
Haustrinae	*Haustrum lacunosum*	Direct	no	no	n.i.	hard	New Zealand	-43	14.95	midpoint
Haustrinae	*Haustrum scobina*	DirectNE	no	no	present	hard	New Zealand	-41.5	16.00	midpoint
Haustrinae	*Haustrum vinosum*	DirectNE	no	no	present	hard	Australia	-37.5	14.00	midpoint
Muricinae	*Bolinus brandaris*	DirectNE	no	no	present	hard	Eastern Atlantic	31.5	18.96	midpoint
Muricinae	*Chicoreus brunneus*	Planktotrophic	yes	yes	absent	hard	Indowest Pacific	7.5	27.00	site of collection
Muricinae	*Chicoreus ramosus*	Planktotrophic	yes	yes	absent	hard	Indowest Pacific	8.5	28.58	site of collection
Muricinae	*Chicoreus torrefactus*	Planktotrophic	yes	yes	absent	hard	Indowest Pacific	7.5	27.00	midpoint
Muricinae	*Hexaplex trunculus*	DirectNE	no	no	present	soft	Eastern Atlantic	37.0	21.00	site of collection
Muricinae	*Murex occa*	Direct	no	no	n.i.	soft	Indowest Pacific	8.0	28.58	site of collection
Muricinae	*Murex trapa*	LecithotrophicNE	yes	no	present	soft	Indowest Pacific	15.0	26.93	midpoint
Muricinae	*Phyllonotus pomum*	DirectNE	no	no	present	hard	Western Atlantic	13.0	27.53	site of collection
Muricopsinae	*Vitularia salebrosa*	Planktotrophic	yes	yes	absent	hard	Eastern Pacific	9.0	28.00	site of collection
Ocenebrinae	*Acanthina monodon*	DirectNE	no	no	present	hard	Eastern Pacific	-34.7	13.02	site of collection
Ocenebrinae	*Acanthinucella paucilirata*	DirectNE	no	no	present	hard	Eastern Pacific	32.5	16.81	site of collection
Ocenebrinae	*Acanthinucella spirata*	DirectNE	no	no	present	hard	Eastern Pacific	39.4	12.49	midpoint
Ocenebrinae	*Ceratostoma rorifluum*	Direct	no	no	n.i.	hard	Eastern Pacific	40.0	14.73	site of collection
Ocenebrinae	*Chorus giganteus*	LecithotrophicNE	yes	no	present	soft	Eastern Pacific	-31.0	15.67	midpoint
Ocenebrinae	*Mexacanthina angelica*	Direct	no	no	n.i.	hard	Eastern Pacific	30.0	23.00	midpoint
Ocenebrinae	*Mexacanthina lugubris*	Direct	no	no	n.i.	hard	Eastern Pacific	28.3	18.83	midpoint

Table S1 (cont.)

Subfamily	Species name	Mode of larval development	Pelagic	Feeding	Nurse eggs	Habitat	Ocean	Latitude (-S)	SST	Point used
Ocenebrinae	*Nucella lamellosa*	Direct	no	no	absent	hard	Eastern Pacific	46.5	11.25	site of collection
Ocenebrinae	*Nucella lapillus*	DirectNE	no	no	present	hard	Eastern Pacific	46.3	12.65	site of collection
Ocenebrinae	*Nucella canaliculata*	DirectNE	no	no	present	hard	Eastern Pacific	44.0	12.47	site of collection
Ocenebrinae	*Ocenebra erinaceus*	DirectNE	no	no	present	hard	Eastern Atlantic	50.3	12.60	site of collection
Ocenebrinae	*Ocinebrina aciculata*	Direct	no	no	n.i.	hard	Eastern Atlantic	41.0	16.00	midpoint
Ocenebrinae	*Urosalpinx cinerea*	Direct	no	no	absent	hard	Western Atlantic	36.8	16.37	midpoint
Ocenebrinae	*Urosalpinx perrugata*	Direct	no	no	n.i.	soft	Western Atlantic	29.0	24.21	site of collection
Ocenebrinae	*Xanthochorus cassidiformis*	Planktotrophic	yes	yes	absent	soft	Eastern Pacific	-41.5	13.13	site of collection
Rapaninae	*Concholepas concholepas*	Planktotrophic	yes	yes	absent	hard	Eastern Pacific	-31.7	13.50	site of collection
Rapaninae	*Dicathais orbita*	Planktotrophic	yes	yes	absent	hard	Southwestern Pacific	-43.0	12.36	site of collection
Rapaninae	*Plicopurpura pansa*	Planktotrophic	yes	yes	absent	hard	Eastern Pacific	10.5	22.50	midpoint
Rapaninae	*Purpura persica*	Planktotrophic	yes	yes	absent	hard	Indowest Pacific	24.5	26.30	midpoint
Rapaninae	*Rapana venosa*	Planktotrophic	yes	yes	absent	soft	Indowest Pacific	41.0	25.00	site of collection
Rapaninae	*Reishia clavigera*	Planktotrophic	yes	yes	absent	hard	Indowest Pacific	0.5	28.53	midpoint
Rapaninae	*Stramonita chocolata*	Planktotrophic	yes	yes	absent	hard	Eastern Pacific	-19.6	13.60	site of collection
Rapaninae	*Stramonita haemastoma*	Planktotrophic	yes	yes	absent	hard	Eastern Pacific	23.5	17.00	site of collection
Rapaninae	*Stramonita rustica*	Planktotrophic	yes	yes	absent	hard	Western Atlantic	25.7	26.93	site of collection
Rapaninae	*Indothais lacera*	Planktotrophic	yes	yes	absent	hard	Indowest Pacific	24.5	26.27	site of collection
Trophoninae	*Trophon geversianus*	DirectNE	no	no	present	hard	Western Atlantic	-47.9	11.00	site of collection

Mode of larval development (NE: nurse eggs), type of development coded as presence of pelagic or feeding larva, presence or absence of nurse eggs (n.i: no information available), type of habitat occupied by adults (coded as soft or hard bottoms), general distribution of the species (ocean), latitude and sea water temperature (SST) of the point used for each species.

,
