# Supplementary material for: Correlated Evolution between Mode of Larval Development and Habitat in Muricid Gastropods
Source: PLoS One. 2014 Apr 8;9(4):e94104. doi: 10.1371/journal.pone.0094104 (PMC3979742; doi:10.1371/journal.pone.0094104)
Supplement: Table S2 — GenBank accession numbers for the species used in the phylogenetic analysis. (PDF) [file pone.0094104.s002.pdf]

**Table S2:** GenBank accession numbers for the species used in the phylogenetic analysis.

| Subfamily | Species with larval development information | Species found in GenBank | COI | 16S RNA | 12S RNA | 28S RNA |
|---|---|---|---|---|---|---|
| ***Outgroup*** | *Buccinum undatum* | *Buccinum undatum* | FN677402 | FN677455 | FN677400 | EU391567 |
| ***Outgroup*** | *Hemifusus tuba* | *Hemifusus tuba* | HQ834069 | JN052953 | HQ833891 | |
| ***Outgroup*** | *Nassarius festivus* | *Nassarius festivus* | HQ834071 | HQ833941 | HQ833893 | |
| ***Outgroup*** | *Conus textile* | *Conus textile* | HQ834089 | HQ833962 | HQ833914 | |
| Ergalataxinae | *Drupella cornus* | *Drupella cornus* | FR853841 | FM999122 | FM999091 | FM999147 |
| Ergalataxinae | *Drupella margariticola* * | *Ergalatax margariticola* | JN053035 | JN052973 | HQ833879 | EU391552 |
| Ergalataxinae | *Drupella rugosa* | *Drupella rugosa* | FR853838 | JN052968 | FR854000 | FR853918 |
| Ergalataxinae | *Tenguella granulata** | *Morula granulata* | JN053032 | JN052972 | HQ833880 | FN677469 |
| Ergalataxinae | *Tenguella marginalba** | *Morula marginalba* | FJ516253 | HE583934 | HE583783 | HE583865 |
| Ergalataxinae | *Tenguella musiva** | *Morula musiva* | GU188266 | GU188165 | HQ833881 | FN677472 |
| Haustrinae | *Bedeva paivae* | *Bedeva paivae/Lepsiella paivae* | FN677412 | FN677437 | FN677387 | FN677466 |
| Haustrinae | *Haustrum lacunosum* | *Haustrum lacunosum* | FN677411 | FN677442 | FN677388 | FN677465 |
| Haustrinae | *Haustrum scobina* | *Haustrum scobina* | | FN677441 | FN677386 | FN677467 |
| Haustrinae | *Haustrum vinosum* | *Haustrum vinosum* | FJ516044 | FN677435 | FN677385 | FN677468 |
| Muricinae | *Bolinus brandaris* | *Bolinus brandaris* | DQ280020 | DQ280052 | | DQ279986 |
| Muricinae | *Chicoreus brunneus* | *Chicoreus brunneus* | GU575371 | FN651899 | FN651857 | FN651965 |
| Muricinae | *Chicoreus ramosus* | *Chicoreus ramosus* | FJ784239 | | | |
| Muricinae | *Chicoreus torrefactus* | *Chicoreus torrefactus* | GU188211 | GU188103 | | HM583730 |
| Muricinae | *Hexaplex trunculus* | *Hexaplex trunculus* | EU391577 | AM712603 | AM712302 | EU391563 |
| Muricinae | *Murex occa* | *Murex occa* | | | | EU391544 |
| Muricinae | *Murex trapa* | *Murex trapa* | GU188199 | GU188090 | HQ833874 | HM583721 |
| Muricinae | *Phyllonotus pomum* | *Phyllonotus pomum* | U86328 | | | |
| Muricopsinae | *Vitularia salebrosa* | *Vitularia salebrosa* | FN651947 | FN651923 | FN651878 | FN651989 |
| Ocenebrinae | *Acanthina monodon* | *Acanthina monodon* | EU391583 | FN677445 | FN677401 | HM486922 |
| Ocenebrinae | *Acanthinucella paucilirata* | *Acanthinucella paucilirata* | AY017490 | | | |
| Ocenebrinae | *Acanthinucella spirata* | *Acanthinucella spirata* | AY017515 | | | |
| Ocenebrinae | *Ceratostoma rorifluum* | *Ceratostoma rorifluum* | JN053041 | JN052974 | | HM583724 |
| Ocenebrinae | *Chorus giganteus* | *Chorus giganteus* | | | | HM486924 |
| Ocenebrinae | *Mexacanthina angelica* * | *Mexacanthina lugubris angelica* | FJ839928 | | | |
| Ocenebrinae | *Mexacanthina lugubris* * | *Mexacanthina lugubris lugubris* | FJ839960 | | | |

**Table S2 (cont.)**

| Subfamily | Species with larval development information | Species found in GenBank | COI | 16S RNA | 12S RNA | 28S RNA |
|---|---|---|---|---|---|---|
| Ocenebrinae | *Nucella lamellosa* | *Nucella lamellosa* | AY445494 | | | |
| Ocenebrinae | *Nucella lapillus* | *Nucella lapillus* | FN651945 | FN651920 | FN651876 | EU391560 |
| Ocenebrinae | *Nucella canaliculata* | *Nucella canaliculata* | AY231233 | | AF076593 | |
| Ocenebrinae | *Ocenebra erinaceus** | *Ocenebra erinacea* | | | | AF327546 |
| Ocenebrinae | *Ocinebrina aciculata* | *Ocinebrina aciculata* | FR851906 | | | |
| Ocenebrinae | *Urosalpinx cinerea* | *Urosalpinx cinerea* | FN677423 | FN677440 | FN677371 | FN677478 |
| Ocenebrinae | *Urosalpinx perrugata* | *Urosalpinx perrugata* | DQ868957 | | | |
| Ocenebrinae | *Xanthochorus cassidiformis* | *Xanthochorus cassidiformis* | FN651948 | FN651924 | FN651879 | FN651990 |
| Rapaninae | *Concholepas concholepas* | *Concholepas concholepas* | JF728889 | HQ260596 | FN677398 | EU391554 |
| Rapaninae | *Dicathais orbita* | *Dicathais orbita* | EU391573 | FN677450 | DQ916434 | EU391553 |
| Rapaninae | *Plicopurpura pansa* | *Plicopurpura pansa* | HE584358 | HE584268 | HE584108 | HE584189 |
| Rapaninae | *Purpura persica** | *Purpura rudolphi* | GU188256 | HQ833968 | HQ833919 | |
| Rapaninae | *Rapana venosa* | *Rapana venosa* | GU188188 | GU188078 | HQ833872 | EU399883 |
| Rapaninae | *Reishia clavigera* | *Reishia clavigera/Thais clavigera* | GU188226 | GU188123 | HQ833876 | HM583734 |
| Rapaninae | *Thaisella chocolata* * | *Thais chocolata* | FR695724 | EU636210 | FR696254 | FR696124 |
| Rapaninae | *Stramonita haemastoma* | *Stramonita haemastoma/ Thais haemastoma* | FR695839 | FM999121 | FR696228 | FR696159 |
| Rapaninae | *Stramonita rustica* | *Stramonita rustica* | FR695847 | HE584303 | FR696214 | FR696135 |
| Rapaninae | *Indothais lacera* | *Indothais lacera/Thais lacera* | KC466632 | HE584243 | HE584080 | HE584166 |
| Trophoninae | *Trophon geversianus* | *Trophon geversianus* | FN651949 | FN651926 | FN651881 | FN651992 |

*Current species name according WoRMS.
